# Performance Impact of Lead‐Free CsSn_0.5_Ge_0.5_I_3_ Based Perovskite Solar Cells with HTL‐Free Incorporation

**DOI:** 10.1002/gch2.202400141

**Published:** 2024-08-28

**Authors:** Md. Shah Alam, Rawdad Nawer Warda, Omi Akter, Dipta Kumar Das

**Affiliations:** ^1^ Department of Electrical & Electronic Engineering University of Chittagong Chittagong 4331 Bangladesh

**Keywords:** CsSn_0.5_Ge_0.5_I_3_, DFT, HTL‐free, lead‐free PSC, SCAPS‐1D

## Abstract

Lead‐containing halide perovskites show promise for solar energy but pose ecological and health risks. To address these, researchers are exploring inorganic binary metal perovskites. This study proposes an eco‐friendly, durable hole transport layer (HTL)‐free design of CsSn_0.5_Ge_0.5_I_3_ with high power conversion efficiency (PCE). Using the SCAPS‐1D simulator, we assessed the efficiency of an HTL‐free planar heterojunction, while the Density Functional Theory (DFT)‐based CASTEP simulator evaluated the optical properties of CsSn_0.5_Ge_0.5_I_3_ in an orthorhombic structure. Key findings highlight enhanced performance under 100 Wm^−2^ AM 1.5G illumination by optimizing absorber layer thickness to 800 nm and reducing defect densities in both the perovskite absorber layer and interfaces to 1 × 10^14^ cm^−3^.Additonally, the effects of different electron transport materials (ETMs), optimization of electron transport layer (ETL) thickness (30‐50 nm), and back contact design improvements were examined. The simulation's results included an increase over the highest values reported in the literature: an open circuit voltage (Voc) of 1.06 V, a short circuit current density (Jsc) of 28.52 mA/cm^2^, a fill factor (FF) of 86.57%, and a PCE of 26.18% for the FTO/Zn_0.875_Mg_0.125_O/CsSn_0.5_Ge_0.5_I_3_/Se perovskite solar cell (PSC). This research provides theoretical insights for developing high‐efficiency power modules without HTLs with significant industrial and research potential.

## Graphical abstract

A lead‐free, safer, eco‐friendly, and durable inorganic photovoltaic solar cells (PSCs) with Hole Transport Layer (HTL) ‐free incorporation pays attention in this study. Noted for high efficiency, **CsSn0.5Ge0.5I3** as an absorber layer simplifies production, enhances stability, and reduces costs, marking its first use as an absorber layer. The illustration demonstrates the envisioned device in conjunction with the crystalline arrangement of the absorbing stratum.

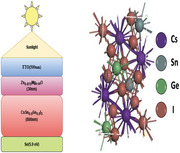

## 1 Introduction

Perovskite is a substance with a crystalline composition according to the formula ABX_3_.^[^ ^1^ ^]^ The perovskite material exhibits remarkable potential for multifaceted utilization, serving not only as a light‐absorbing layer but also as an electron/hole transport layer. Their functionality originates from their strong radiation absorption and reflection, high charge carrier mobility, extended lifetime, considerable depth of carrier length, and significant carrier diffusion interval^[^ ^2^, ^3^ ^]^ The perovskite‐based photovoltaic devices have exhibited a significant enhancement in their overall PCE from 3.8% to 27.3% which positioned them as a potential competitor for the upcoming era of single junction solar cells, with projections predicting that they will outperform traditional silicon solar cells in the future.^[^ ^3^, ^4^, ^5^, ^6^ ^]^ A recent study on hot carrier perovskite solar cells achieved 27.3% efficiency using a sulfur‐modified phthalocyanine (Pc) hole transport layer.^[^ ^6^ ^]^ However, it is associated with concentrated photovoltaics which require a specialized system to manage the increased heat and light intensity. Another study shows that a perovskite solar cell achieved 30.9% efficiency using thermally evaporated sublimed C_60_ as an ETL. However, as C_60_ material can clump together during thermal evaporation, it needs further purification by sublimation to get 99.95% purity, increasing the complexity and expenses.^[^ ^7^ ^]^ Several investigations on tin‐based (Sn) perovskites, such as FASnI_3_ (HC(NH_2_)_2_SnI_3_), MASnI_3_ (CH_3_NH_2_SnI_3_), and CsSnI_3_, have determined that perovskites based on organic cations (FA, MA) exhibited inherent low stability.^[^ ^8^ ^]^ As a result, Cesium (Cs) based cation perovskite became the preferred applicant for the position.^[^ ^9^ ^]^ A planar heterojunction design has been employed to produce a lead‐free substitute in Sn‐based perovskites.^[^ ^10^ ^]^ When exposed to oxygen, electronics based on tin can undergo an oxidation process called Sn^2+^ to Sn^4+^, which results in a degradation of the device's characteristics and can lead to instability. Min et al. proffered the concept of alloying Cesium Tin Iodide (CsSnI_3_) with Germanium ion (Ge^2+^) to effectuate the formation of the exceedingly stable and air‐tolerant binary metal CsSn_0.5_Ge_0.5_I_3_ perovskite configuration. This modification improves the perovskite solar cell's structural stability due to its Goldschmidt tolerance factor of 0.94 and Octahedral factor of 0.4.^[^ ^11^ ^]^

A general perovskite solar cell (PSC) consists of an ETL (Electron Transport Layer) and an HTL (Hole Transport Layer). These layers efficiently obstruct the migration of electrons or holes from the absorber to the front or back contact, while facilitating the transport of the generated electrons and holes to the metal contacts. However, manufacturing fault‐free multilayered PV devices is challenging. In addition, defects at the interface of ETL or HTL/perovskite might facilitate carrier recombination and lower the overall architectural equilibrium of a solar cell. HTL and ETL work together to help the cell collect photons as efficiently as possible.^[^ ^12^ ^]^ At this time, the majority of lab‐scale high‐efficiency PSCs use noble metal as the back electrode and organic hole transport layers (HTLs). Both of these components contribute to the instability of the PSCs as well as the high fabrication costs, which prevents further commercialization that is unnecessary.^[^ ^13^, ^14^, ^15^ ^]^ A PSC without a compact layer demonstrates that ETL and HTL are not required to achieve high device efficiency. Various findings on HTL‐free and ETL‐free perovskite solar cells reveal that the lack of both interfaces does not interfere with device function. These studies demonstrate that devices can work well even without an HTL/ETL interface.^[^ ^16^ ^]^ HTL‐free photovoltaic solar cells (PSCs) have earned significant interest due to the restrictions posed by metal electrodes and organic HTLs due to their streamlined production techniques, outstanding stability, and low fabrication prices.^[^ ^17^, ^18^ ^]^ Adil Sunny et al proposed a Pb and HTL‐free CH_3_NH_3_SnI_3_‐based PSC employing CH_3_NH_3_SnI_3_ as an absorber layer and TiO_2_ as ETL.^[^ ^19^ ^]^ By changing variables like thickness, temperature, defect density, resistance, etc., the numerical simulation is used to figure out how well the suggested device works (**Table** **1**).

**Table 1:** Comprehensive overview of the related literature.

References	Absorber layer	Stability	Efficiency	Advantages	Disadvantages
[1]	CsPbI_3_	Poor stability	Not specified	Chemically stable. Susceptible to thermal stressing.	Poor phase stability Lead toxicity.
[7]	p‐i‐n PSC (thermally evaporated C60)	Good stability	30.9%	Suitable for tandem solar cells. Mass reproducibility.	Cost‐effective. Complex Procedure. Repeated evaporation process.
[20]	MAPbI_3_	Susceptible to moisture, temperature UV degradation.	24.2%	Cost effective. High absorption. Tunable band gap.	Lead toxicity. Instability. Degradation issues.
[21]	CsGeI_3_	Poor due to sensitivity to oxygen	Not specified	Lead‐free Ideal band gap	Low efficiency Sensitivity to Oxygen. Poor stability.
[21]	Cs_3_Sb_3_I_9_	Poor performance	Not specified	Lead‐free	Poor performance Large band gap unsuitable for solar devices.
[22]	Cs_2_TiBr_6_	Good stability	11.49%	Higher stability. Toxicity free	Less data. Poor efficiency. Sensitive.

In our proposed structure, the Cesium Tin‐Germanium Iodide (CsSn_0.5_Ge_0.5_I_3_) that serves as the primary active material in our perovskite solar cell is shown. We have presented an HTL‐free perovskite solar cell to assess its PCE, J_SC_, V_OC_, and FF. We have suggested using a structure composed of FTO/ETL/CsSn_0.5_Ge_0.5_I_3_/Au. CsSn_0.5_Ge_0.5_I_3_ demonstrates high efficiency as an environmentally friendly solar cell through the selection of verified composition parameters, improved fabrication techniques, and simpler procedures. By varying the materials used for the ETL and Back contact, we can determine a structure with optimal efficiency. We also examined the optical features of CsSn_0.5_Ge_0.5_I_3_ via first‐principles calculations based on density functional theory (DFT). The optical properties are computed in terms of absorption coefficient, refractive index, and electrical conductivity.

## 2 Simulation Procedure

The investigation of the HTL‐free planar heterojunction architecture, comprising an FTO/ETL/CsSn_0.5_Ge_0.5_I_3_/Metal back contact, was performed using the Solar Cell Capacitance Simulator (SCAPS‐1D). The development of the SCAPS‐1D simulator, which fulfills the demands of device modeling, was initiated by the Department of Electronic and Information Systems (ELIS) at the University of Gent.^[^ ^23^ ^]^ The analysis of the optical properties of CsSn_0.5_Ge_0.5_I_3_ was performed based on DFT. Computational investigation of optical properties (absorption, reflectivity, conductivity) has been conducted through the utilization of the Cambridge Serial Total Energy Package (CASTEP), a quantum mechanical code that was developed by Payne and his colleagues during the 1990s.^[^ ^24^ ^]^

**Figure 1:**
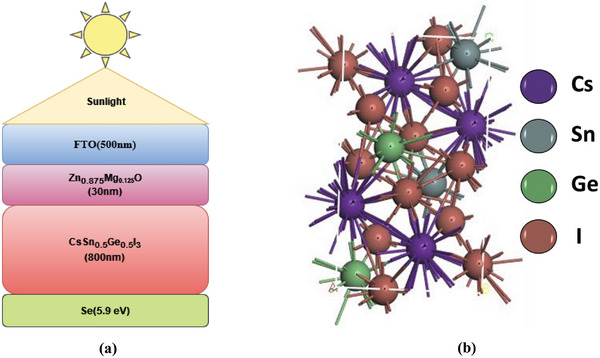
a) Proposed device configuration b) Crystal structure of absorber layer.

**Figure** **1**a illustrates our proposed HTL‐free heterojunction perovskite solar consisting of the FTO/Zn_0.875_Mg_0.125_O/CsSn_0.5_Ge_0.5_I_3_/Se. We have used an interface defect density, Interface Defect Layer (IDL) at ETL/CsSn_0.5_Ge_0.5_I_3_ (perovskite layer). Figure 1b depicts the P1 phase of the CsSn_0.5_Ge_0.5_I_3_ structure. The crystal and surface structure of CsSn_0.5_Ge_0.5_I_3_ are illustrated in the subsequent figure. The analysis of the optical properties of the bulk is conducted through the utilization of Density Functional Theory (DFT) and band structure. Our chosen configuration for the bulk form of CsSn_0.5_Ge_0.5_I_3_ is the orthorhombic arrangement, which displays the 62 Pnma symmetry of the space group.

**Figure 2:**
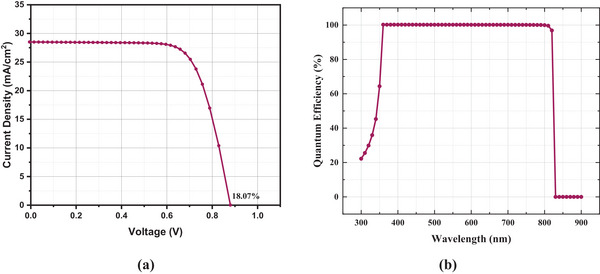
a) *J–V* curve and b) Quantum efficiency versus Wavelength curve of FTO/Zn_0.875_Mg_0.125_O/CsSn_0.5_Ge_0.5_I_3_/Se structure.

In our proposed cell, the neutral types of defects are mentioned for each slab up to 0.6 eV of applied energy. This defect has a normal Gaussian distribution with a standard deviation value of 0.1 eV. The electrons and holes' thermal velocities are 10^7^ cm s^−1^. In addition to selenium (Se) back contact with a work function of 5.9 eV, we employed FTO with a work function of 4.4 eV. We used a 1 Ω cm^2^ series resistance and 5 000 Ω cm^2^ shunt resistance, with a constant operating temperature of 300K and 1 000 Wm^−2^ illumination at AM 1.5G. The presented J‐V and QE plots, depicted in **Figure** **2**, were obtained by utilizing the initial values furnished within **Table** **2** and **3**.

**Table 2:** Simulation Parameters for PSC Structure in FTO and Absorber Layer.

Properties	FTO	CsSn_0.5_Ge_0.5_I_3_	IDL
Thickness, d (nm)	500	200(varied)	10
E_g_ (eV)	3.5	1.5	1.85
χ(eV)	4	3.9	4.15
Ɛ_r_ (eV)	9	28	6.6
N_C_ (cm^−3^)	2.2 × 10^18^	3.1 × 10^18^	2.0 × 10^19^
N_V_ (cm^−3^)	1.8 × 10^19^	3.1 × 10^18^	2.0 × 10^19^
µ_e_ (cm^2^/V‐s)	20	974	15
µ_p_ (cm^2^/V‐s)	20	213	2
N_D_ (cm^−3^)	2 × 10^19^	0	1.0 × 10^17^
N_A_ (cm^−3^)	0	1 × 10^14^	0
N_t_ (cm^−3^)	1 × 10^15^	1 × 10^16^(varied)	1.0 × 10^15^
References	[25]	[25]	[22]

**Table 3:** Input parameters for various ETL materials.

Properties	Zn_0.875_Mg_0.125_O	ZnO	SnO_2_	TiO_2_	PCBM	WS_2_	CdS
Thickness (nm)	30 (varied)	50	30	150	50	50	50
E_g_ (eV)	3.48	3.3	3.5	3.2	2	1.8	2.4
χ (eV)	4.37	4	4	4	3.9	3.95	4.8
Ɛ_r_ (eV)	9	9	9	10	3.9	13.6	10
N_V_ (1 cm^−3^)	9 × 10^17^	1 × 10^19^	2.2 × 10^16^	1 × 10^20^	2.5 × 10^21^	2.2 × 10^17^	1.9 × 10^19^
N_C_ (1 cm^−3^)	9 × 10^16^	1 × 10^19^	2.2 × 10^17^	1.5 × 10^20^	2.5 × 10^21^	2.2 × 10^16^	2.2 × 10^18^
µ_e_(cm^2^ /Vs)	50	100	200	20	0.2	100	100
µ_p_(cm^2^ /Vs)	20	100	80	10	0.2	100	25
N_D_ (cm^−3^)	1 × 10^16^	5 × 10^17^	1 × 10^17^	0	2.93 × 10^17^	1 × 10^18^	1 × 10^18^
N_A_ (cm^−3^)	0	0	0	5 × 10^19^	0	0	0
N_T_ (cm^−3^)	1 × 10^15^	1 × 10^15^	1 × 10^15^	1 × 10^15^	1 × 10^15^	1 × 10^15^	1 × 10^15^
References	[26]	[25, 27]	[28]	[27, 29]	[27]	[27]	[26]

Our cell has extremely high photoelectric conversion efficiency, as further evidenced by the QE diagram. The validity of this research is shown by the fact that the performance predicted by the initial parameters is essentially in line with the results of experiments performed on perovskite solar cells based on CsSn_0.5_Ge_0.5_I_3_.^[^ ^11^ ^]^

## 3 Results & Analysis

### 3.1 Optoelectronic Properties Analysis of CsSn_0.5_Ge_0.5_I_3_

The optical properties of the specimens are explicated in correlation to variables encompassing absorption, refractive index, and electrical conductivity. Each of these derived parameters is contingent upon the energy magnitude of electromagnetic radiation.

**Figure 3:**
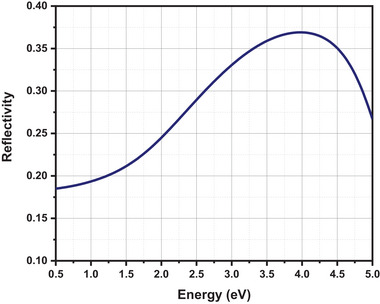
Optical reflectivity curve of CsSn_0.5_Ge_0.5_I_3_.

A crucial aspect of solar cells is the interaction between the optical reflectivity and the energy of the incident light as well as the electronic band structure. High reflectivity in solar cells decreases absorption and increases reflection. The absorption spectroscopy in **Figure** **3** shows an inverse relationship between absorption and light reflection. The material's surface and volume contribute to reflection, especially in materials that diffuse light. The graphs indicate that the reflectivity in the visible region (1.77 to 3.12 eV) ranges from 0.22 to 0.34. These values indicate that the material absorbs a substantial amount of incident light and only 22% to 34% of reflected back.^[^ ^21^ ^]^

**Figure 4:**
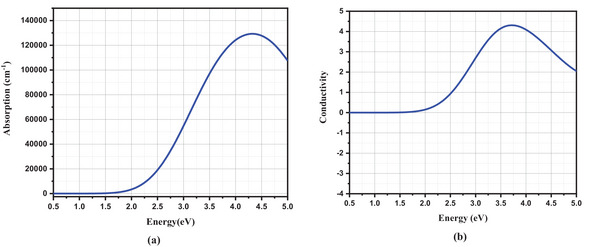
Optical Properties of absorber layer a) Absorption b) Conductivity.

The fluctuation in the absorption coefficient of CsSn_0.5_Ge_0.5_I_3_ is determined by the Energy Band Structure (EBS) of the underlying substances and the energy level of the incident light. Electrons gain energy and depart from the valence band to the conduction band when light passes through the material. This absorbed energy creates the absorption peak. The absorption coefficient determines the better efficiency for storing Solar Energy in solar cells. The computed absorption spectra, as depicted in **Figure** **4**a, reveal a range between 2.5 eV to more than 5 eV compared to a significant peak in the same region. However, it is noteworthy that the highest peak occurs in the 4.21 eV light energy range, which falls within the UV spectrum. Therefore, it is also used as a UV absorber.^[^ ^30^ ^]^ Figure 3 shows that CsSn_0.5_Ge_0.5_I_3_ has a higher reflectance in the UV area. Since visible light absorbs between 1.63 and 3.26 eV, it exhibits efficient absorption characteristics at both visible and UV wavelengths. This material works efficiently as an absorber in solar cells due to its moderate absorption and low reflection in the visible range.^[^ ^31^ ^]^

The optical conductivity of CsSn_0.5_Ge_0.5_I_3_ is significantly influenced by the electronic structure and characteristics of this material. It is observed that CsSn_0.5_Ge_0.5_I_3_ exhibits significant light absorption in the visible range. The maximum conductivity of CsSn_0.5_Ge_0.5_I_3_ is observed within the energy range of 3.48–3.92 eV. As illustrated in Figure 4b, CsSn_0.5_Ge_0.5_I_3_ exhibits an optical conductivity of 4.32(1/fs). The graph showing conductivity is strongly linked to the absorption spectra, with high absorption indicating high conductivity. This material has the highest conductivity peak in the UV region and an optical conductivity of 0.91(1/fs) to 3.41(1/fs) in the visible region, showing efficient absorption of visible light.^[^ ^32^ ^]^

### 3.2 Optimization of Perovskite Layer Thickness

The thickness of the absorber layer holds a significant effect on the thin‐film solar cell. The optimal thickness is crucial to ensure the highest level of photon absorption and charge carrier pair generation in perovskite's absorber layer.

**Figure 5:**
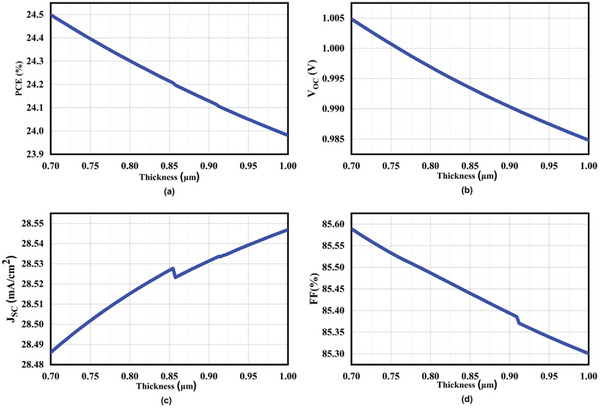
Characteristics of Absorber layer thickness a) PCE b) Voc c) Jsc d) FF.

We have effectively optimized the thickness of the perovskite absorber layer, resulting in an increase from 0.7 to 1 µm. **Figure** **5** illustrates the efficiency of CsSn_0.5_Ge_0.5_I_3_ at 0.7 µm has the maximum value of 24.50%, while the value of CsSn_0.5_Ge_0.5_I_3_ at 1 µm has the lowest value of 23.98%. With an increase in material thickness, cell performance degrades. However, there is a swift increase in J_SC_ until reaching 0.85 µm, followed by a slight decrease and subsequently another increase with further thickness augmentation. The reason for the slight decrease and subsequent increase in current density is that, when the absorber layer thickness surpasses 0.85 micrometers, the process of charge recombination initiates at the midpoint of the material, specifically before reaching the electrical contacts. This phenomenon results in a saturation effect occurring at increased thickness levels.^[^ ^33^ ^]^ The explanation for the increment in J_SC_ is the augmented absorption of photons in this specific range.^[^ ^28^ ^]^ When the thickness increases, it becomes more visible, which is why recombination is detrimental to the collection of charge carriers and V_OC_ starts to plummet.^[^ ^34^ ^]^ We can see the fill factor for CsSn_0.5_Ge_0.5_I_3_ reduces when the thickness expands. As the voltage applied to the diode increases, there is a consequent reduction in the electrically charged surface present in the absorber layer, ultimately resulting in a significant decline in the FF.^[^ ^22^, ^35^ ^]^

### 3.3 Electron Transport Layer Optimization

By preventing photogenerated holes and reducing the likelihood of electron‐hole recombination, the ETL makes it easier for electrons to travel through the device. An efficient electron transport layer (ETL) exhibits characteristics such as enhanced electron mobility, optimized thickness and doping concentration, and a suitable bandgap^[^ ^28^ ^]^

**Figure 6:**
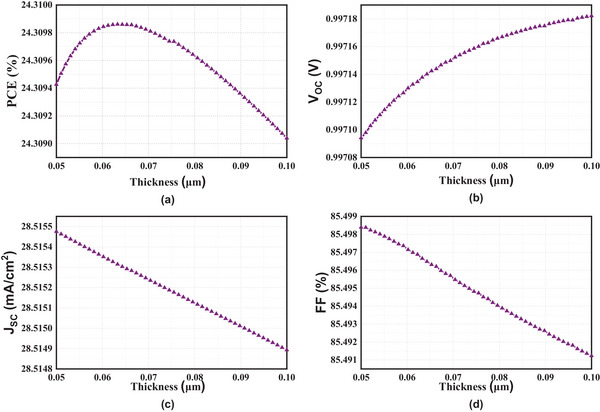
Cell parameters for the ETL thickness variations a) PCE b) Voc c) Jsc d) FF.

We have used PCBM, SnO_2_, TiO_2_, ZnO, CdS, Zn_0.875_Mg_0.125_O for this study. The conduction band offset (CBO) values associated with SnO_2_, TiO_2_, ZnO, CdS, and WS_2_ are −0.1, −0.1, −0.2, −0.4, and −0.5 eV, respectively.^[^ ^36^ ^]^ As the CBO is negative for all layers, this indicates that they are all potential ETL candidates. **Figure** **6** displays a reduction in effectiveness, short circuit current, and fill factor with an increase of the Zn_0.875_Mg_0.125_O layer thickness. The cell's overall efficiency is suffering as a direct result of the development of bigger pinholes and a lumpy surface. Additionally, the electron‐hole pair recombination increased as the thickness increased. This resulted in a significant increase in series resistance, which in turn decreased the J_SC_ and the efficiency of the material.^[^ ^37^ ^]^

Thinner materials exhibit enhanced performance due to reduced minority carrier concentration and decreased rates of radiative, non‐radiative, and auger recombination. Consequently, it is necessary to keep the thickness of the ETL to a minimum in order to achieve maximum efficiency.^[^ ^38^ ^]^ However, it cannot be any thinner than 0.062 µm due to manufacturing difficulties.

### 3.4 Absorber Defect Density Optimization

A comprehensive investigation is required to improve solar cell efficiency because every material has its own unique set of deep‐defect levels. We have analyzed the absorber defect densities ranging from 10^14^ to 10^20^.

**Figure 7:**
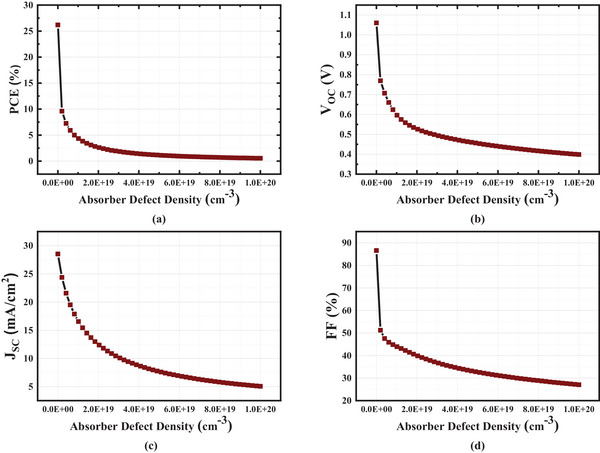
Characteristics of Absorber Defect Density a) PCE b) Voc c) Jsc d) FF.

A linear relationship between absorber defect density and performance characteristics is shown in **Figure** **7**. At 10^14^ cm^−3^ density, the PCE is 26.18%, while at 10^20^ cm^−3^ density, it is 0.54%. An increase in defects in a solar cell leads to an increment in the collective electron‐hole recombination current, which subsequently reduces the performance of the cell.^[^ ^23^ ^]^ The solar cell's characteristics gradually declined as the density of absorber flaws increased, from 1014 to 1020 cm^−3^. The fabrication of a material with an extraordinarily low density of flaws poses significant challenges. This is caused by the increased number of recombination centers in the absorber layer, which reduces the lifespan of the hole and electron carriers. As a result, the lifetimes of holes and electrons are lowered. This is owing to the concern that increasing the defect density will raise the recombination rate.^[^ ^20^ ^]^ Therefore, we have opted to maintain the absorber defect density at 10^14^ cm^−3^.

### 3.5 Interface Defect Density Optimization

The defect density at the Electron Transport Material's (ETM) interface was discovered to have a substantial impact on the solar cell's efficiency. The defect density had no substantial effect on the performance characteristics of the PSC within the layer of the Hole Transport Material (HTM) and absorber interface.^[^ ^39^ ^]^ In the current arrangement of the PSC without an HTL, there exists a singular connection that links the ETL and the light‐absorbing material. The level of imperfections at the ETL/light‐absorbing material interface greatly impacts both the long‐term durability and efficiency of the photovoltaic device.^[^ ^27^ ^]^

**Figure 8:**
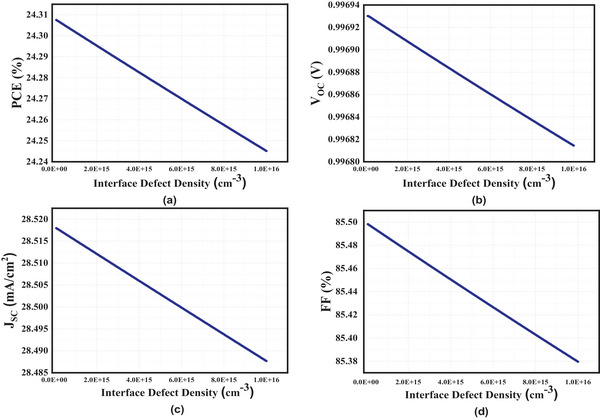
Interface defect density Characteristics a) PCE b) Voc c) Jsc d) FF.

We evaluated the device's efficiency by increasing the intensity of defects at the ETL/absorber layer interface to a maximum of 10^16^ from 10^14^ cm^−3^. The augmentation in interface defect density is accompanied by a linear decrease in all performance parameters, as illustrated in **Figure** **8**. The PCE exhibits a rate of 24.30% at a defect density of 10^14^ cm^−3^, whereas a slightly lower rate of 24.245% is observed at 10^16^ cm^−3^. Furthermore, the current density at the lower defect density is measured to be 28.52 mA cm^−^^2^, while at the higher defect density, it is recorded as 28.49 mA cm^−^^2^. An increased density of interfacial defects results in an escalation of charge carrier recombination at the interface, consequently leading to a degradation in cell performance.^[^ ^40^ ^]^

### 3.6 Effect of Shunt and Series Resistance

Both the shunt and series resistances have an important influence on the device's performance since they control the contours and gradients of the *J–V* characteristics. A low R_shunt_ might result in photovoltage loss as well as an increase in photocurrent collection. In addition, R_series_ has a significant influence on FF and short‐circuit current values. Low R_series_ and high R_shunt_ values are commonly regarded as critical for highly efficient devices.^[^ ^41^, ^42^ ^]^

**Figure 9:**
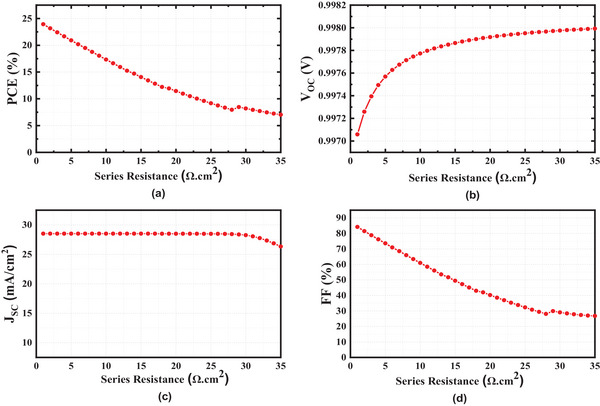
Characteristics of series resistance a) PCE b) Voc c) Jsc d) FF.

To comprehend the impact of R_Series_ on absolute device characteristics, we varied their values from 1 to 35 Ω cm^2^. The changes in PCE, V_OC_, J_SC_, and FF of the device are graphically depicted in **Figure** **9**. The PCE has an ultimate value of 23.924% at 1 Ω cm^2^ and a minimum value of 7.02479% at 35 Ω cm^2^. Because ohmic loss is indicated by series resistance in perovskite solar cells, an expansion of series resistance causes an increase in base doping density, which in turn causes a growth in the total resistivity of the semiconductor material. As a direct consequence of this, the number of defects increases, which in turn gradually brings the PCE, JSC, and FF down.^[^ ^41^ ^]^ With an increase in the series resistance, the voltage experiences a gradual and continuous escalation. When the series resistance exceeds 30 Ω cm^2^, J_SC_ begins a gradual but consistent decline. An increase in this series resistance results in a gradual increment of the defect number which in turn leads to reductions in PCE, J_SC_, and FF.^[^ ^41^ ^]^ So, series resistance is kept minimum for the better efficiency of the cell. Here the optimized series resistance is 1 Ωcm^2^.

**Figure 10:**
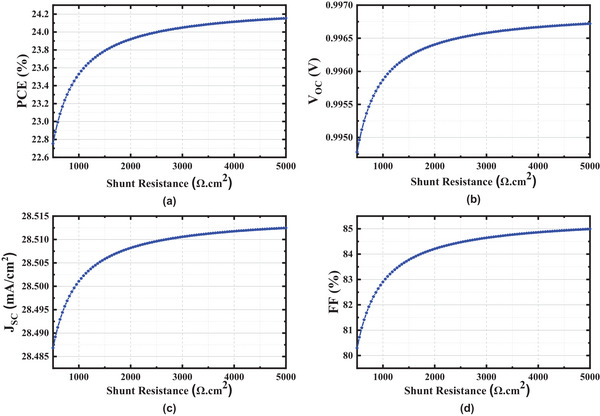
Characteristics of shunt resistance a) PCE b) Voc c) Jsc d) FF.

We have investigated the device performance by varying R_shunt_ from 500 to 5000 Ω cm^2^. From the results presented in **Figure** **10**, it is evident that the PCE is observed to be at its maximum when the shunt resistance is 5000 Ω cm^2^. Conversely, the efficiency is observed to be at its minimum when the shunt resistance is 500 Ω cm^2^. Due to the increase in shunt resistance, electron‐hole recombination in volume, exterior, and possible short circuits at the solar cell's junction occurs. When the shunt resistance is considerable, the leakage current is low in the structured device, and when the resistivity is small, the leakage current is high.^[^ ^43^ ^]^ So, the shunt resistance was kept at a maximum.

### 3.7 Back Contact Optimization

In the HTL‐free PSC, the back contact assumes a crucial function. Since there is no HTL layer, the direct attachment to the absorber layer makes it highly impactful. Au, Pd, Ni, Pt, and Se are the only available back contact metals for solar cells. Cu and Ag solar cells with short circuits have shown linear current density‐voltage curves with no adjustable characteristics.^[^ ^44^ ^]^

**Figure 11:**
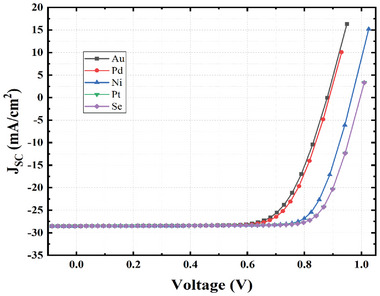
Optimization of alternative back contacts.

From **Figure** **11** we can see the optimization of the alternative back contacts. The graph illustrates that the suggested design enhances efficiency with the work function of 5.40 eV. According to the simulation, both Pt and Se exhibit the same level of efficiency, with Pt having an energy of 5.6 eV and Se having an energy of 5.9 eV. We have decided to use selenium (Se) as the back contact rather than platinum (Pt) because platinum's price is so outrageously high.

### 3.8 Effect of Operating Temperature

**Figure 12:**
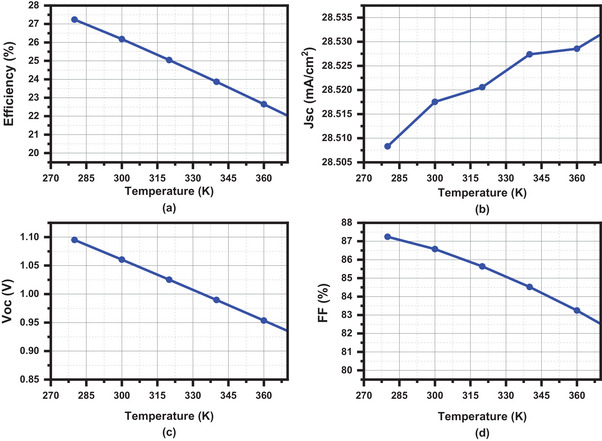
Characteristics of operating temperature a) Efficiency b) J_SC_ c) V_OC_ d) FF.

Temperature is a crucial factor in solar cell performance. To ensure the stability of the HTL‐free CsSn_0.5_Ge_0.5_I_3_‐based PSC, the effect of temperature in the proposed solar cell was tested at temperatures ranging from 280 to 370K in 15K increments. The operation of the device is impacted by temperature since all performance metrics are dependent on temperature. In **Figure** **12**, it can be observed that because of the drop in band gap energy, J_SC_ somewhat rises with temperature.^[^ ^45^ ^]^ The graph demonstrates a notable decline in the efficiency, V_OC_, and FF of the proposed device as the temperature rises. With the increase in temperature, the electrons exhibit reduced stability due to their enhanced energy levels, hindering their recombination with holes. Consequently, this leads to a rise in carrier concentration and lattice vibration, while decreasing the bandgap energy and saturation current, ultimately causing a decrease in the overall performance of the device.^[^ ^19^, ^46^ ^]^

### 3.9 Optimized Device

**Figure 13:**
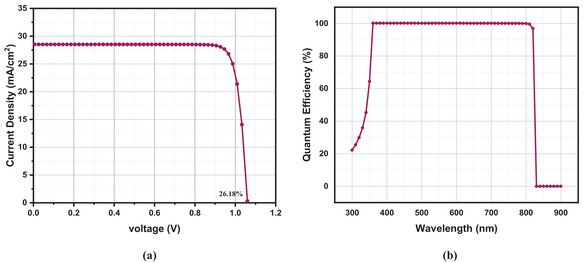
a) *J–V* curve and b) QE curve of FTO/Zn_0.875_Mg_0.125_O/CsSn_0.5_Ge_0.5_I_3_/Se structure.

In our proposed design, we have configured a CsSn_0.5_Ge_0.5_I_3_‐based HTL‐free PSC which is capable of delivering remarkable photovoltaic performance. The findings of our study have opened the door for the development of a CsSn_0.5_Ge_0.5_I_3_‐based HTL‐free power supply that is less complicated, less expensive, more efficient, and more stable. **Table** **4** compares the performance of PSCs utilizing CsSn_0.5_Ge_5_I_3_ as an absorber layer, with and without the use of an HTL. The comparison reveals that our proposed structure exhibits superior efficiency. **Figure** **13** displays the optimized J‐V and QE curves of our device.

**Table 4:** Comparison of HTL‐based PSC and HTL‐free PSC.

Design structure	J_SC_ [mA cm^−2^]	V_OC_ [V]	FF [%]	PCE [%]
HTL based^[^ ^44^ ^]^	27.05	0.87	79.25	18.79
HTL‐free (Proposed design)	**28.52**	**1.06**	**86.57**	**26.18**

**Table** **5** presents a comparative analysis of various solar cell structures incorporating the CsSn_0.5_Ge_0.5_I_3_ absorber layer. This comparison highlights the performance differences and efficiencies among the evaluated configurations. Our proposed absorber layer exhibits superior efficiencies compared to other absorber layers.

**Table 5:** Comparison of CsSn_0.5_Ge_0.5_I_3_ (absorber layer) based PSC.

Structure	J_SC_ [mA cm^−2^]	V_OC_ [V]	FF [%]	PCE [%]
FTO/TiO_2_/CsSn_0.5_Ge_0.5_I_3_/SMe‐TATPyr/Au^[^ ^47^ ^]^	22.24	1.10	81.89	20.22
FTO/PCBM/CsSnGeI_3_/Spiro‐OMeTAD/Au^[^ ^48^ ^]^	25.912	0.729	68.566	12.956
Au/ CsSn_0.5_Ge_0.5_I_3_/ZnO‐Np/FTO^[^ ^49^ ^]^	17.2450	0.9968	82.14	14.12
Au/ CuI/ CsSn_0.5_Ge_0.5_I_3_/ZnO‐Np/FTO^[^ ^49^ ^]^	24.2167	1.2011	84.2	24.5
Au/ Spiro‐OMeTAD/ CsSn_0.5_Ge_0.5_I_3_/ZnO‐Np/FTO^[^ ^49^ ^]^	24.2176	1.2107	83.42	24.46
FTO/PC_60_BM/ CsSn_0.5_Ge_0.5_I_3_/Spiro‐OMeTAD/Au^[^ ^50^ ^]^	28.70	1.115	87.86	18.13
FTO/CeO_x_/ CsSn_0.5_Ge_0.5_I_3_/PTAA/Au^[^ ^51^ ^]^	25.80	1.17	80.33	24.20
**FTO/Zn_0.875_Mg_0.125_O/CsSn_0.5_Ge_0.5_I_3_/Se (This Work)**	**28.52**	**1.06**	**86.58**	**26.18**

**Table** **6** shows the comparison of different HTL‐free structures. From the table, we can see that the proposed structure has the best efficiency of 26.18% among all the research works.

**Table 6:** Efficiency comparison of HTL‐free structures.

Structure (ETL/PAL)	J_SC_ [mA cm^−2^]	V_OC_ [V]	FF [%]	PCE [%]
TiO_2_/CH_3_NH_3_PbI_3_/C^[^ ^29^ ^]^	31.93	0.98	84.34	24.31
WO_3_/CH_3_NH_3_PbI_3_^[^ ^27^ ^]^	24.66	1.06	59.15	15.54
SnO_2_/MAPbI_3_^[^ ^52^ ^]^	Varies	Varies	Varies	22
TiO_2_/(MA:FA)PbI_3_^[^ ^53^ ^]^	9.58	0.77	54	4
Zn(O_0.3_S_0.7_)/FASnI_3_^[^ ^54^ ^]^	24.25	1.07	87.87	22.72
Al_2_O_3_/MAPbBr_3_^[^ ^55^ ^]^	2.7	1.35	0.55	2.02
**Our Proposed structure**	**28.52**	**1.06**	**86.58**	**26.18**

## 4 Conclusion

This study reports on the development, using the SCAPS‐1D simulation tool, of an HTL‐free PSC with the structure of FTO/Zn_0.875_Mg_0.125_O/CsSn_0.5_Ge_0.5_I_3_/Se. The attainment of the proposed PSC is studied using numerical reconstruction to determine how changes to the ETL material, thickness, interface defect density, absorber defect density, series and shunt resistances, and back contact metal affect the device's overall functionality. The results show that our proposed HTL‐free PSC structure is capable of delivering remarkable photovoltaic performance. In addition, series resistance resulting from bulk and interfaces also contributes to poor device performance. Studies have demonstrated that utilizing back metal contact materials possessing a high work function has the potential to enhance the performance characteristics of a cell. We have obtained notable results, including a V_OC_ value of 1.06 V, a J_SC_ value of 28.52 mA cm^−2^, an FF value of 86.58%, and a PCE value of 26.18%, by integrating the absorber layer CsSn_0.5_Ge_0.5_I_3_ and optimizing the ETL.

## Conflict of Interest

The authors declare no conflict of interest.

## Data Availability

The data that support the findings of this study are available in the supplementary material of this article.
